# MimicrEE2: Genome-wide forward simulations of Evolve and Resequencing studies

**DOI:** 10.1371/journal.pcbi.1006413

**Published:** 2018-08-16

**Authors:** Christos Vlachos, Robert Kofler

**Affiliations:** Institut für Populationsgenetik, Vetmeduni Vienna, Veterinärplatz, Wien, Austria; University of Technology Sydney, AUSTRALIA

## Abstract

Evolve and Resequencing (E&R) studies allow us to monitor adaptation at the genomic level. By sequencing evolving populations at regular time intervals, E&R studies promise to shed light on some of the major open questions in evolutionary biology such as the repeatability of evolution and the molecular basis of adaptation. However, data interpretation, statistical analysis and the experimental design of E&R studies increasingly require simulations of evolving populations, a task that is difficult to accomplish with existing tools, which may i) be too slow, ii) require substantial reformatting of data, iii) not support an adaptive scenario of interest or iv) not sufficiently capture the biology of the used model organism. Therefore we developed MimicrEE2, a multi-threaded Java program for genome-wide forward simulations of evolving populations. MimicrEE2 enables the convenient usage of available genomic resources, supports biological particulars of model organism frequently used in E&R studies and offers a wide range of different adaptive models (selective sweeps, polygenic adaptation, epistasis). Due to its user-friendly and efficient design MimicrEE2 will facilitate simulations of E&R studies even for small labs with limited bioinformatics expertise or computational resources. Additionally, the scripts provided for executing MimicrEE2 on a computer cluster permit the coverage even of a large parameter space. MimicrEE2 runs on any computer with Java installed. It is distributed under the GPLv3 license at https://sourceforge.net/projects/mimicree2/.

This is a *PLOS Computational Biology* Software paper.

## Introduction

The Evolve and Resequencing (E&R) approach is a powerful tool for studying adaptation at a genome-wide scale [1, 2]. The advent of next generation sequencing (NGS) made it feasible to study genomic changes occurring in populations subject to any form of artificial or natural selection [1, 2]. Usually allele frequency changes are monitored, for example by sequencing pools of populations (Pool-Seq [3]), but it is also feasible to study changes of the haplotype structure [4]. Monitoring the genomic response to selection ultimately promises to shed light on some major open questions in evolutionary biology such as the genetic basis of complex traits [5], the distribution of fitness effects [6], the mode of adaptation (e.g. polygenic adaptation vs. selective sweeps [7, 8]) and the repeatability of evolution [9].

However E&R studies increasingly require genome-wide forward simulations of adapting populations, where especially three key challenges stand out:

First, E&R studies come at a considerable cost, both in terms of money and time. For example studying adaptation in Drosophila for 60 generations may take up to two years and require several thousand Dollars of sequencing cost [2, 10]. In case of a suboptimal experimental design it may be impossible to answer the pertinent research questions and the invested resources may have been wasted. It is therefore of considerable interest to evaluate the power of an experimental design before embarking on a costly E&R study. Computer simulations may, for example, help to identify the optimal number of replicates, generations of selection and starting haplotypes [11, 12, 13].

Second, many different test statistics for identifying selected loci in E&R studies have been suggested [5, 9, 14, 15, 16]. Computer simulations can help to identify the strength and weaknesses of these different statistics [2, 17]. Simulations have, for example, shown that time-series based test statistics could increase the power to identify selected loci in E&R studies [14, 15]. Additionally, the validation of novel methodological approaches, for example the reconstruction of haplotypes from E&R data [4], requires computer simulations.

Finally several E&R studies have found an unexpected genomic response to selection. For example, Kosheleva and Desai [8] observed that experimentally evolving yeast populations responded to selection at the genomic level for about 240 generations but no further response was found during the following 720 generations. The authors suggested that a quantitative trait model involving many loci of small effect in combination with diminishing returns epistasis could generate this pattern. Computer simulations will allow to test whether a proposed model, such as this, could account for the observed data.

In summary, it is likely that computer simulations will be an integral part of future E&R studies, be it in study design or interpretation of the data. To aid researchers in these tasks we developed MimicrEE2 (mimicry of experimental evolution) a tool for fast genome-wide forward simulations of evolving populations.

## Design and implementation

**Fig 1:**
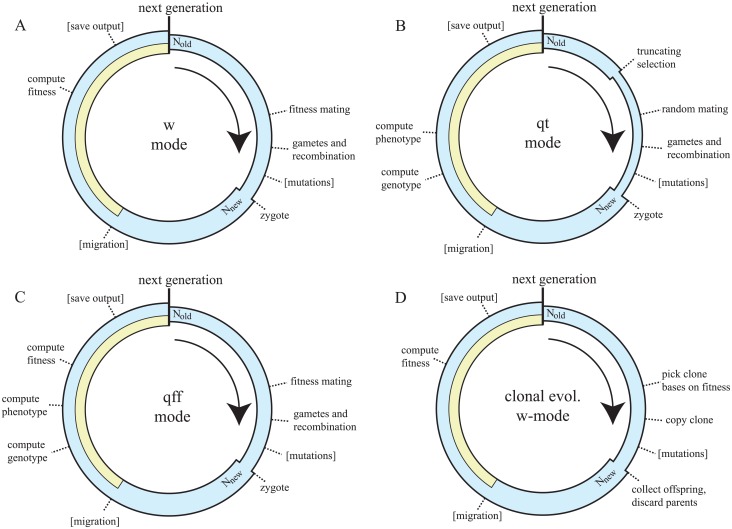
Flow diagrams showing the order of events occurring at each generation during simulations with MimicrEE2. A separate diagram is shown for each model of selection (i.e. mode’s) supported by MimicrEE2. A) At the w-mode the fitness of each individual is directly computed from the selection coefficients of the SNPs present in the genome. The mating success of individual scales with fitness. B) With the qt-mode, MimicrEE2 first computes the phenotypic values for each individual based on the effect sizes of the SNPs and some environmental variance. Then it performs truncating selection, where the individuals with the most pronounced phenotypic values are culled. C) During the qff-mode, MimicrEE2 computes the phenotypic values of a quantitative trait and maps these values to fitness using a fitness function (e.g.: a Gaussian fitness function for stabilizing selection). D) Events occurring during clonal evolution using the w-mode as example. Most importantly, clones do not mate but generate identical copies of themselves (with the exception of *de novo* mutations). In the flow diagram’s yellow indicates migrants and the width of the circles indicates the population size. Optional events are shown in square brackets.

MimicrEE2 is a user-friendly tool for individual based forward simulations of evolving populations. It uses a discrete time model and allows simulating haploid as well as diploid organism. As an important feature MimicrEE2 enables usage of available genomic resources such as haplotypes or recombination maps. MimicrEE2 supports three different models of selection (Fig 1):

- w-mode (w is a widely used symbol for fitness): fitness of individuals is directly computed from the selection coefficients of SNPs
- qt-mode: a quantitative trait is simulated and phenotypically extreme individuals are truncated
- qff-mode: a quantitative trait is mapped to fitness using a fitness function

For several reasons MimicrEE2 is especially suitable for genome-wide forward simulations of E&R studies. First, it supports biological particularities of model organism commonly used in E&R studies. MimicrEE2 allows to simulate haploid and diploid organism, different forms of reproduction (e.g. males and females in Drosophila, different ratios between males and hermaphrodites in Caenorhabditis), variable rates of self-fertilization, clonal evolution, hemizygous sex chromosomes and sex specific recombination maps (e.g. Drosophila males do not recombine). Second, MimicrEE2 supports many different models of adaptation, like classic selected loci, complex epistasis between pairs of loci (fitness may be provided for all combinations of genotypes), selection on a quantitative trait, truncating selection, stabilizing selection, diminishing returns epistasis, disruptive selection, directional selection and adaptation to a moving optimum. Third, MimicrEE2 is comparatively user friendly (no programming skills are necessary) and enables the convenient use of available genomic resources such as haploytpe data, recombination maps and known positions of causative loci. Fourth, MimicrEE2 supports multi-threading and we provide scripts that allow running MimicrEE2 on computer clusters (Apache Spark spark.apache.org). This allows simulating even powerful experimental designs (e.g. large population size and many replicates) in a time effective manner. Finally the output of MimicrEE2 (sync, fasta) is compatible with many downstream tools frequently used for analyzing E&R data, such as tools for identifying selected loci (PoPoolation2, poolSeq, CLEAR, BBGP [18, 17, 15, 14]), reconstructing haplotypes [4] and simulating reads (e.g. ART [19]).

MimicrEE2 is implemented in Java and does not require installation of any libraries or tools, hence it is platform independent and runs on any computer with Java installed (v8 or higher; tested with macOS and Linux). To install MimicrEE2 it is solely necessary to download the java archive file (jar; see manual https://sourceforge.net/p/mimicree2/wiki/Manual/). As input MimicrEE2 requires haplotypes for a population and the recombination rate. Haplotypes need to be provided as nucleotides (A,T,C,G), which simplifies conversion from commonly used file formats such as vcf files. An arbitrary number of chromosomes may be provided. MimcrEE2 converts the haplotypes into a bitarray (0,1), therefore solely biallelic SNPs may be used. A recombination rate may be provided for arbitrary sized windows. For each window the mean number of cross overs is computed using Haldane’s mapping function [20] and the number of cross over events is drawn from a Poisson distribution. A random position within the window is picked for each cross over event. At each generation MimicrEE2 performs the following steps in the given order, where details may vary among the modes (Fig 1): i) truncating selection is performed (if applicable), ii) mate pairs are formed, iii) gametes are generated based on cross over events and random assortment of chromosomes iv) mutations are introduced into the gametes (optional) v) zygotes are formed and a novel population is generated vi) migrants are introduced into the population (optional) vii) the genotype, phenotype and fitness of the individuals is computed and viii) the output is stored (optional). For details see the manual (https://sourceforge.net/p/mimicree2/wiki/Manual/).

## Results

MimicrEE2 can be used to i) evaluate the power of different designs of E&R studies, ii) assess the performance of diverse statistical approaches and iii) predict the genomic response to selection under a given model/hypothesis.

**Fig 2:**
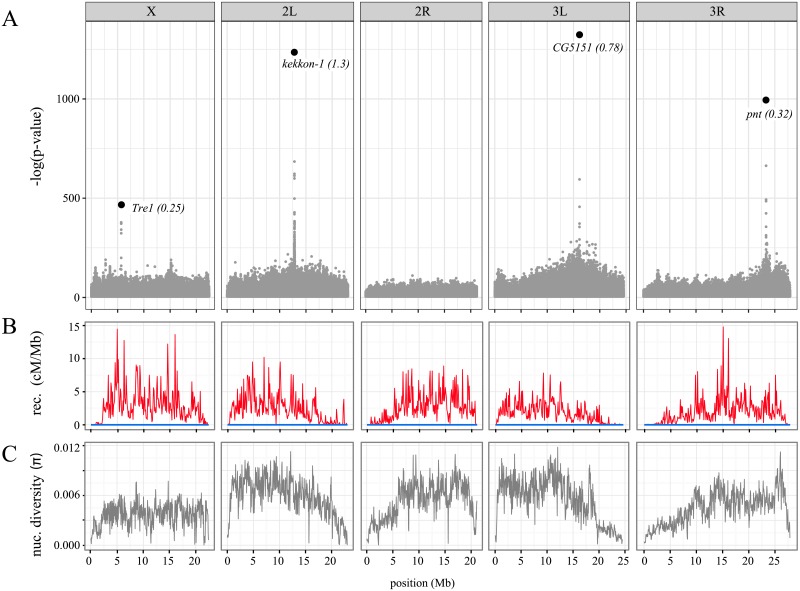
Simulation of truncating selection for starvation resistance in *D. melanogaster*. A) Manhattan-plot showing the significance (cmh-test) of allele frequency differences between the founder and evolved populations, which were subject to truncating selection for 40 generations (10 replicates). Four loci in genes known to contribute to starvation resistance were picked as targets of selection (big black dots, gene names in italics) and an effect size was assigned to each (in brackets). A hemizygous X-chromosome was simulated in males. B) We used a different recombination rate for females (red) and males (blue). C) Nucleotide diversity of the 205 DGRP haplotypes used as founder population.

To illustrate the utility of MimicrEE2 we tested whether it is feasible to identify loci contributing to starvation resistance in Drosophila with a simple truncating selection experiment. Unraveling the genetic basis of complex traits, such as starvation resistance, is considered to be a key challenge for biology in the 21^st^ century [21, 22]. This example also serves to illustrate a major advantage of MimicrEE2, i.e. the user-friendly design which enables convenient usage of available genomic resources. We used 205 haplotypes from the DGRP lines (Drosophila Genome Reference Panel; Freeze 2.0) [23], the recombination rate of *D. melanogaster* [24] and introduced beneficial alleles into four genes known to confer starvation resistance in Drosophila [25]. We used a female to male ratio of 50:50, a hemizygous X-chromosome in males, no recombination in males and simulated truncating selection for 10 replicates and 40 generations, with 80% of the most starvation resistant individuals surviving truncation. Finally we identified the selected loci with PoPoolation2 (cmh-test [18]), directly using the output of MimicrEE2 as input in PoPoolation2. We found that with the simulated experimental design the four targets of selection yield distinct peaks that may be readily identified (Fig 2A). All data and instructions necessary to reproduce this experiment can be found at https://sourceforge.net/p/mimicree2/wiki/BioExample/.

The performance of different experimental designs or test statistics may be evaluated using diagnostic tools such as receiver-operating characteristic (ROC) plots [26] that contrast the true-positive rate (TPR) with the false-positive rate (FPR). To illustrate this we simulated another truncating selection experiment using a slightly more complex architecture of the quantitative trait (50 causative loci). We used several different truncating selection regimes (95%, 80%, 60%, 40%, 20%, 5% of individuals surviving truncation) and repeated the experiment ten times to obtain estimates for the error bars (in total 100 simulations for each condition: 10 experiments with 10 replicates). The population size was 205 and the number of generations 40. Based on the resulting ROC curves we found that truncating selection retaining 95% of the phenotypic most pronounced individuals yielded the highest power to identify the causative variants (S1A Fig). Similarly it is possible to evaluate the suitability of different test statistics for identifying the causative loci (S1B Fig).

Simulations also enable predicting the genomic response under different adaptive models such as stabilizing selection or diminishing returns epistasis (S2 and S3 Figs). Comparison of the observed genomic response to the simulated one enables to assess whether a proposed adaptive scenario could account for the observed data. For example, in a recent work MimicrEE2 was used to test whether selection on a polygenic trait could explain an observed pattern of genetic redundancy, where putatively selected loci only respond in a subset of the replicates [27]. Multiple walkthrough’s for different evolutionary scenarios can be found at https://sourceforge.net/p/mimicree2/wiki/Home/#walkthrough.

## Comparison to other tools

In contrast to its predecessor MimicrEE [11], MimicrEE2 implements several novel features, most notably support for a quantitative trait model, sex, sex chromosomes, migration and *de novo* mutations (Table 1).

**Table 1:** Comparison of tools for genome-wide forward simulations of evolving populations. MimicrEE1 (mim1) [11], MimicrEE2 (mim2, this study), forqs [28], quantiNemo (qNemo) [29], SLiM2 [30], FFPopSim [31].

feature	mim2	mim1	forqs	qNemo	SLiM2	FFPopSim^6^
run time in hours	0.93	0.92	72 (1.81)^5^	6.9	1.01	13.8
required RAM in GB	4.7	4.7	28 (15)^5^	61	2.4	23
quantitative traits	+	-	+	+	o^1^	o^1^
selection coefficients	+	+	+	-	+	+
ploidy (d: diploid, h: haploid)	h/d	d	d	h/d	h/d	h
*de novo* mutations / migration	+/+	-/-	+/+	+/+	+/+	+/o^1^
variable recombination map	+	+	+	+	o^1^	-
sex (♀, ♂, ⚥) / hemizygous sex chromosomes	+/+	-/-	+/-	+/-	+/+	+/-
direct usage of genomic resources^2^	+	+	-	-	-	-
complex epistasis^3^	+	-	-	+	o^1^	+
diminishing returns epistasis	+	-	-	-	o^1^	o^1^
truncating selection (ts) / temporarily variable ts	+/+	-/-	+/-	-/-	o/o^1^	o/o^1^
disruptive / stabilizing selection	+/+	-/-	-/+	-/+	o/o^1^	o/o^1^
adaptation to a moving optimum	+	-	o^4^	+	o^1^	o^1^
gene-environment interactions / spatial model	-/-	-/-	-/-	-/-	+/+	-/-
multi-threading / support for computer cluster	+/+	+/-	-/-	-/-	-/-	-/-
output compatible with E&R tools (sync, fasta)	+	o	-	-	o^1^	o^1^

^1^ requires substantial coding in Eidos or Python, such as implementation of a file parser

^2^ does not require conversion of genotypes into binary (01) or concatenation of chromosomes into one superscaffold

^3^ fitness values may be provided for all combinations of genotypes at pairs of loci

^4^ the effect size of QTLs may vary, not the position and shape of the fitness function

^5^ in brackets: haplotypes are requested as output

^6^ features are referring to the haploid_highd class which allows genome-wide simulations (>20 loci)

Several other tools for genome-wide forward simulations have been developed such as forqs [28], quantiNemo [29], SLiM2 [30] and FFPopSim [31]. To evaluate the performance with E&R data, we performed with each tool the same truncating selection experiment for starvation resistance as described above (Table 1; S1 Text). In cases where truncating selection was not supported we performed neutral simulations (quantiNemo, MimicrEE). As output we requested the allele frequencies. We found that MimicrEE2 was fast and requires little memory (Table 1). Note that we used 8 cores for MimicrEE and MimicrEE2 while we only used a single core for the other tools (multi-threading is not supported by other tools; Table 1). On a per-CPU basis SLiM2 was faster but we consider the actual execution time (i.e. the waiting time) as more important benchmark. Interestingly forqs performance increased when haplotypes instead of allele frequencies are requested as output (Table 1).

SLiM2 [30] is notable as its specifically developed programming language Eidos allows to simulate a wide range of different evolutionary scenarios including spatial models and gene environment interactions (Table 1). However, using some features may require substantial programming skills in Eidos. This raises the difficult question as to which extent a feature is actually supported if it largely needs to be implemented by the user. For example usage of a variable recombination map requires implementing a file parser. We thus opted to indicate “intermediate support” for features that could in principle be simulated but will require substantial coding. The same holds for FFPopSim which requires programming skills in Python (Table 1).

We conclude that of the existing tools MimicrEE2 is best suited for simulating E&R studies as it i) is the fastest tool ii) requires little memory iii) supports the convenient usage of available genomic resources iv) directly supports a wide range of adaptive models and v) is compatible with downstream tools for the analysis of E&R data (Table 1).

## Validation

**Fig 3:**
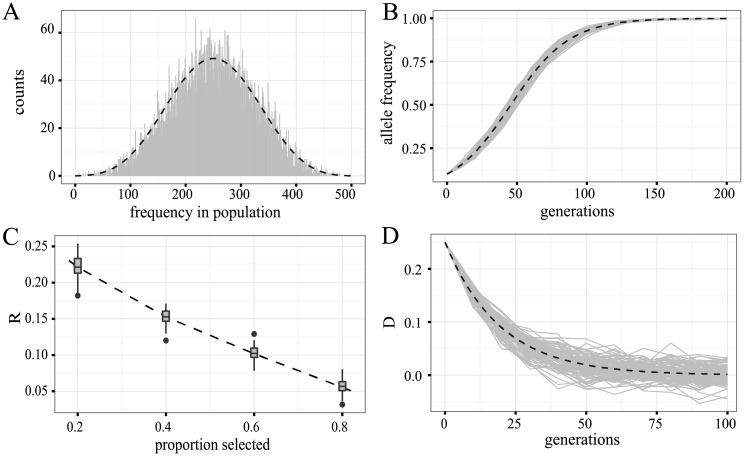
Validation of MimicrEE2. A) Allele frequency distribution of 10.000 SNPs with an initial frequency 0.5 after 50 generations of genetic drift (*N* = 250) compared to theoretical expectations (dashed line). B) Trajectories of 50 selected loci (grey lines; *s* = 0.1, *h* = 0.5) compared to theoretical expectation (dashed line) C) Response to selection (R; box plots based on 100 replicates) of a quantitative trait (*QTLs* = 10, *h*^2^ = 0.5) compared to theoretical expectations (dashed line). D) Decay of linkage disequilibrium between two initially linked loci (*D* = 0.25) due to recombination (*r* = 0.05). We simulated 100 replicates (grey lines) and show theoretical expectations (dashed line).

The main evolutionary forces that shape the frequency of SNPs in E&R studies are genetic drift, selection and recombination. Here, we validated the correct implementation of these forces in MimicrEE2. To test if genetic drift was correctly modeled we simulated 10,000 unlinked loci with a starting allele frequency of 0.5 in a population of size *N* = 250. We performed neutral simulations for 50 generations. We computed the expected allele frequencies using the binomial formula and a Markov Chain model [32]. We found that the obtained allele frequency distribution closely follows the theoretical expectations (Fig 3; Chi-squared test; *χ*^2^ = 482.64; *df* = 500; *p* = 0.70).

Next we tested whether selection was correctly modeled. We simulated codominant loci (*h* = 0.5) having a selective advantage of *s* = 0.1 and a starting allele frequency of 0.1. We used 50 replicates, a population size of *N* = 10.000 and performed forward simulations for 200 generations. Theoretical expectations were derived using the equation $p_{t}=p_{t-1}\left(p_{t-1}W_{AA}+q_{t-1}W_{Aa}\right)/\bar{w}$, where *p*_*t*_ is the allele frequency of the next generation, *p*_*t*−1_ the allele frequency of the previous generation and *W*_*AA*_, *W*_*Aa*_, *W*_*aa*_ the fitness of the genotypes [32]. At each 10^*th*^ generation we compared the obtained allele frequencies to the theoretically expected ones and did not find any significant deviations (Fig 3B; twenty t-tests; *p* > 0.07).

To test whether selection on quantitative traits was correctly implemented we relied on the breeder’s equation (*R* = *h*^2^*S* [33]), which permits to calculate the response to selection (*R*) based on the selected individuals (*S*) and the heritability of a trait (*h*^2^). We simulated 10 QTLs with starting allele frequency 0.5 in a population of *N* = 1000. The heritability was *h*^2^ = 0.5 and 100 simulations were performed for each of the following fractions of selected individuals: 0.2, 0.4, 0.6 and 0.8; The expected response to selection agrees with the observed one (Fig 3C; four *χ*^2^ tests, *df* = 1, *p* > 0.67). Finally we tested whether recombination was modeled correctly by tracing the decay of linkage disequilibrium (LD) between two loci for 100 generations. The alleles at these loci were initially completely linked (*D* = 0.25) and at a frequency of 0.5. We used a recombination rate between the loci of *r* = 0.05 and a population size of *N* = 1000. Theoretical expectations were calculated using the equation *D*_*t*_ = *D*_0_(1 − *c*)^*t*^, where *D*_*t*_ is the LD at the given generation (*t*), *D*_0_ the LD at the starting population and *c* the recombination rate [33]. At each 10^*th*^ generation we compared the observed to the expected LD and did not find any significant deviation (Fig 3D; ten t-tests *p* > 0.09).

To ensure correct behavior of the components of MimicrEE2 we implemented more than 200 unit tests (JUnit 4.12 junit.org/junit4/) that may be executed by the user. More details and further validations can be found at https://sourceforge.net/p/mimicree2/wiki/Home/#validation.

## Availability and future directions

MimicrEE2 is implemented in Java and distributed under the GPLv3 at https://sourceforge.net/projects/mimicree2/. For a detailed manual and a walkthrough with sample data sets see https://sourceforge.net/p/mimicree2/wiki/Home/. A detailed validation of MimicrEE2 can be found at https://sourceforge.net/p/mimicree2/wiki/Home/#validation. For future versions we consider implementing additional fitness functions and to output linkage disequilibrium between pairs of SNPs.

## Supporting information

S1 Fig ROC curve showing the performance of different experimental designs and test statistics. (PDF)

S2 Fig Manhattan plot showing the genomic response to stabilizing selection. (PDF)

S3 Fig Manhattan plot showing the genomic response to diminishing returns epistasis. (PDF)

S1 Text Detailed descriptions of the simulated evolutionary scenarios, the parameters and configuration files used for the different tools, and of the validation of MimicrEE2. (PDF)

## References

[1] Long A, Liti G, Luptak A, Tenaillon O. Elucidating the molecular architecture of adaptation via Evolve and Resequence experiments. Nature Reviews Genetics. 2015;16(10):567–82. 10.1038/nrg3937 26347030 PMC4733663

[2] Schlötterer C, Kofler R, Versace E, Tobler R, Franssen SU. Combining experimental evolution with next-generation sequencing: a powerful tool to study adaptation from standing genetic variation. Heredity. 2015;114:431–440. 10.1038/hdy.2014.86 25269380 PMC4815507

[3] Schlötterer C, Tobler R, Kofler R, Nolte V. Sequencing pools of individuals-mining genome-wide polymorphism data without big funding. Nature Reviews Genetics. 2014;15(11):749–763. 10.1038/nrg3803 25246196

[4] Franssen SU, Barton NH, Schlötterer C. Reconstruction of haplotype-blocks selected during experimental evolution. Molecular Biology and Evolution. 2017;34(1):174–184. 10.1093/molbev/msw210 27702776

[5] Turner TL, D A, Andrew S, Fields T, Rice WR, Tarone AM. Population-Based Resequencing of Experimentally Evolved Populations Reveals the Genetic Basis of Body Size Variation in Drosophila melanogaster. PLoS Genetics. 2011;7(3):e1001336 10.1371/journal.pgen.1001336 21437274 PMC3060078

[6] Desai MM. Statistical questions in experimental evolution. Journal of Statistical Mechanics: Theory and Experiment. 2013;2013(01):P01003 10.1088/1742-5468/2013/01/P01003

[7] Pritchard JK, Di Rienzo A. Adaptation—not by sweeps alone. Nature reviews Genetics. 2010;11(10):665–7. 10.1038/nrg2880 20838407 PMC4652788

[8] Kosheleva K, Desai MM. Recombination alters the dynamics of adaptation on standing variation in laboratory yeast populations. Molecular Biology and Evolution. 2017;35:180–201. 10.1093/molbev/msx278 PMC5850740 29069452

[9] Remolina SC, Chang PL, Leips J, Nuzhdin SV, Hughes KA. Genomic basis of aging and life-history evolution in Drosophila melanogaster. Evolution; international journal of organic evolution. 2012;66(11):3390–403. 10.1111/j.1558-5646.2012.01710.x 23106705 PMC4539122

[10] Orozco-Terwengel P, Kapun M, Nolte V, Kofler R, Flatt T, Schlötterer C. Adaptation of Drosophila to a novel laboratory environment reveals temporally heterogeneous trajectories of selected alleles. Molecular Ecology. 2012;21(20):4931–4941. 10.1111/j.1365-294X.2012.05673.x 22726122 PMC3533796

[11] Kofler R, Schlötterer C. A Guide for the Design of Evolve and Resequencing Studies. Molecular biology and evolution. 2014;31(2):474–483. 10.1093/molbev/mst221 24214537 PMC3907048

[12] Kessner D, Novembre J. Power Analysis of Artificial Selection Experiments Using Efficient Whole Genome Simulation of Quantitative Traits. Genetics. 2015;199(4):991–1005. 10.1534/genetics.115.175075 25672748 PMC4391575

[13] Baldwin-Brown JG, Long AD, Thornton KR. The Power to Detect Quantitative Trait Loci Using Resequenced, Experimentally Evolved Populations of Diploid, Sexual Organisms. Molecular biology and evolution. 2014;31:1040–55. 10.1093/molbev/msu048 24441104 PMC3969567

[14] Topa H, Jónás Á, Kofler R, Kosiol C, Honkela A. Gaussian process test for high-throughput sequencing time series: Application to experimental evolution. Bioinformatics. 2015;31(11):1762–1770. 10.1093/bioinformatics/btv014 25614471 PMC4443671

[15] Iranmehr A, Akbari A, Schlötterer C, Bafna V. CLEAR: Composition of likelihoods for evolve and resequence experiments. Genetics. 2017;206(2):1011–1023. 10.1534/genetics.116.197566 28396506 PMC5499160

[16] Terhorst J, Schlötterer C, Song YS. Multi-locus Analysis of Genomic Time Series Data from Experimental Evolution. PLoS Genetics. 2015;11(4):e1005069 10.1371/journal.pgen.1005069 25849855 PMC4388667

[17] Taus T, Futschik A, Schlötterer C. Quantifying Selection with Pool-Seq Time Series Data. Molecular Biology and Evolution. 2017;34:3023–3034. 10.1093/molbev/msx225 28961717 PMC5850601

[18] Kofler R, Pandey RV, Schlötterer C. PoPoolation2: identifying differentiation between populations using sequencing of pooled DNA samples (Pool-Seq). Bioinformatics (Oxford, England). 2011;27(24):3435–6. 10.1093/bioinformatics/btr589 PMC3232374 22025480

[19] Huang W, Li L, Myers JR, Marth GT. ART: a next-generation sequencing read simulator. Bioinformatics (Oxford, England). 2012;28(4):593–4. 10.1093/bioinformatics/btr708 PMC3278762 22199392

[20] Haldane JBS. The combination of linkage values and the calculation of distances between the loci of linked factors; 1919.

[21] Stapley J, Reger J, Feulner PGD, Smadja C, Galindo J, Ekblom R, et al Adaptation genomics: the next generation. Trends in Ecology & Evolution. 2010;25(12):705–712. 10.1016/j.tree.2010.09.002 20952088

[22] Losos JB, Arnold SJ, Bejerano G, Brodie ED III, Hibbett D, Hoekstra HE, et al Evolutionary Biology for the 21st Century. PLoS Biology. 2013;11(1):e1001466 10.1371/journal.pbio.1001466 23319892 PMC3539946

[23] Mackay T, Richards S, Stone E, Barbadilla A, Ayroles J, Zhu D, et al The *Drosophila melanogaster* genetic reference panel. Nature. 2012;482(7384):173–178. 10.1038/nature10811 22318601 PMC3683990

[24] Comeron JM, Ratnappan R, Bailin S. The Many Landscapes of Recombination in *Drosophila melanogaster*. PLoS Genetics. 2012;8(10):e1002905 10.1371/journal.pgen.1002905 23071443 PMC3469467

[25] Harbison ST, Yamamoto AH, Fanara JJ, Norga KK, Mackay TFC. Quantitative Trait Loci Affecting Starvation Resistance in *Drosophila melanogaster*. Genetics. 2004;166(4):1807–1823. 10.1534/genetics.166.4.1807 15126400 PMC1470806

[26] Hastie T, Tibshirani R, Friedman J. The elements of statistical learning; 2nd edition vol. 1 Springer series in statistics New York; 2008.

[27] Barghi N, Tobler R, Nolte V, Jaksic AM, Mallard F, Otte K, et al Polygenic adaptation fuels genetic redundancy in Drosophila. bioRxiv. 2018; p. 332122. 10.1371/journal.pbio.3000128 PMC6375663 30716062

[28] Kessner D, Novembre J. Forqs: Forward-in-time simulation of recombination, quantitative traits and selection. Bioinformatics. 2014;30(4):576–577. 10.1093/bioinformatics/btt712 24336146 PMC3928523

[29] Neuenschwander S, Hospital F, Guillaume F, Goudet J. quantiNemo: An individual-based program to simulate quantitative traits with explicit genetic architecture in a dynamic metapopulation. Bioinformatics. 2008;24(13):1552–1553. 10.1093/bioinformatics/btn219 18450810

[30] Haller BC, Messer PW. SLiM 2: Flexible, interactive forward genetic simulations. Molecular Biology and Evolution. 2017;34(1):230–240. 10.1093/molbev/msw211 27702775

[31] Zanini F, Neher R. FFPopSim: an efficient forward simulation package for the evolution of large populations. Bioinformatics. 2012;28(24):3332–3333. 10.1093/bioinformatics/bts633 23097421 PMC3519462

[32] Gillespie JH. Population genetics: a concise guide. JHU Press; 2010.

[33] Falconer DS. Introduction to quantitative genetics. Oliver And Boyd; Edinburgh; London; 1960.

