# Supplementary material for: MimicrEE2: Genome-wide forward simulations of Evolve and Resequencing studies
Source: PLoS Comput Biol. 2018 Aug 16;14(8):e1006413. doi: 10.1371/journal.pcbi.1006413 (PMC6112681; doi:10.1371/journal.pcbi.1006413)
Supplement: S1 Fig — (PDF) [file pcbi.1006413.s001.pdf]

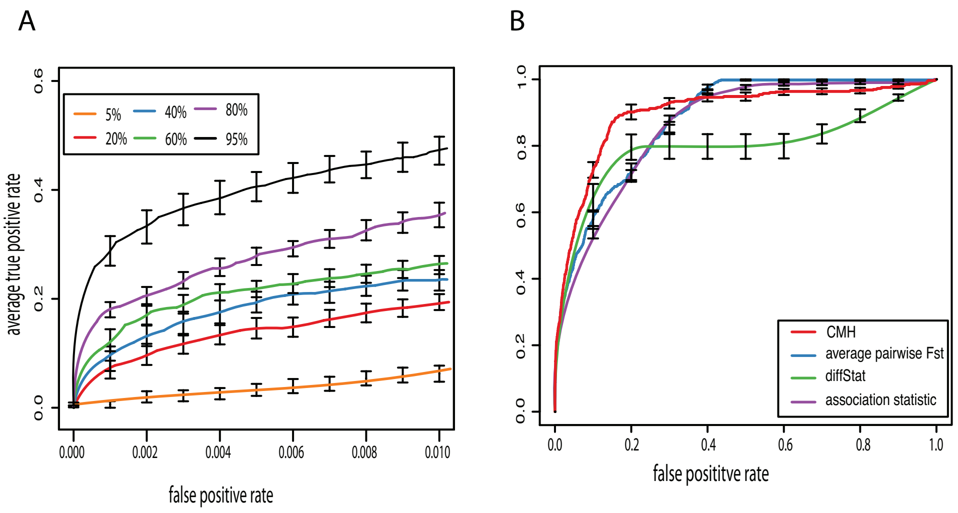

Figure 1: Genome-wide forward simulations can be used to evaluate the power of different selection regimes (a) and test statistics (b) with ROC curves. The more the ROC curve is bent towards the left upper corner the better the performance of an approach a) Performance of different truncating selection regimes. Different fractions of individuals with the highest phenotypic values were selected. b) Performance of four test statistics for identifying selected loci in E&R studies.
