# Supplementary material for: MimicrEE2: Genome-wide forward simulations of Evolve and Resequencing studies
Source: PLoS Comput Biol. 2018 Aug 16;14(8):e1006413. doi: 10.1371/journal.pcbi.1006413 (PMC6112681; doi:10.1371/journal.pcbi.1006413)
Supplement: S2 Fig — (PDF) [file pcbi.1006413.s002.pdf]

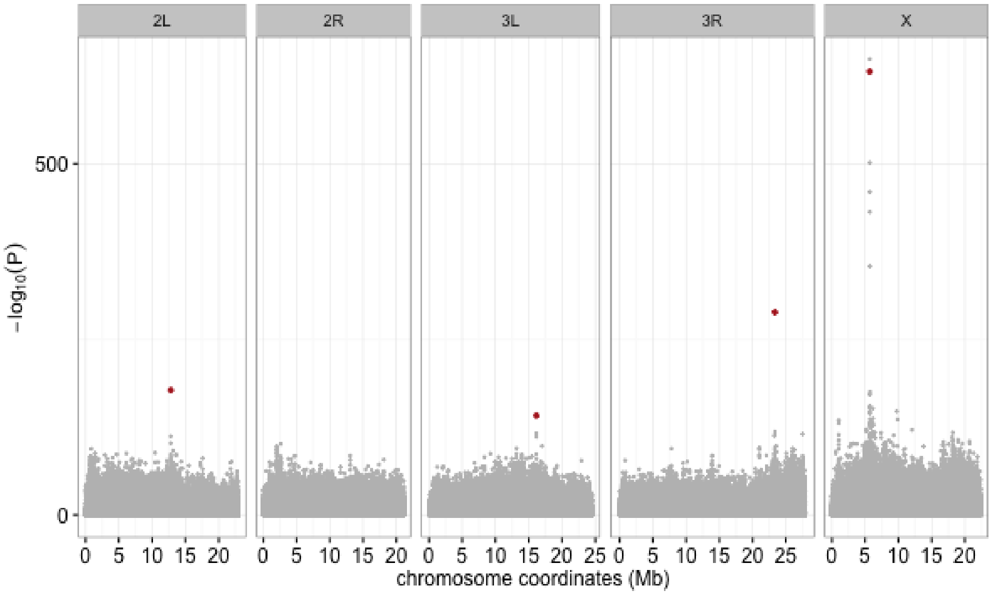

Figure 2: Manhattan plot for a simulated E&R study with 4 QTLs evolving under stabilizing selection.
