# Supplementary material for: MimicrEE2: Genome-wide forward simulations of Evolve and Resequencing studies
Source: PLoS Comput Biol. 2018 Aug 16;14(8):e1006413. doi: 10.1371/journal.pcbi.1006413 (PMC6112681; doi:10.1371/journal.pcbi.1006413)
Supplement: S1 Text — (PDF) [file pcbi.1006413.s004.pdf]

Supplementary material and methods

Performance of selection regimes and test statistics

We evaluated the performance of six different truncating selection regimes. We used the 205 haplotypes from the DGRP lines (Freeze 2.0 [Mackay et al., 2012]) and the recombination rates for *D. melanogaster*. We randomly picked 50 QTLs (effect size= 0.25 for 25 QTL, 0.78 for 15 QTL and 1.3 for 10 QTL) and used a heritability of $h^2 = 1$. In each generation 95%, 80%, 60%, 40%, 20%, 5% of the individuals with the largest phenotypic values survived truncating selection. We performed truncating selection for 40 generations and used 10 replicates. To obtain estimates of the error bars we picked 10 independent sets of QTLs for each selection regime (in total $6*10*10 = 600$ simulations) The cmh-test [Kofler et al., 2011] was used to detect the selected loci.

To evaluate the suitability of different test-statistic for identifying selected loci in E&R studies we compared the performance of the cmh-test [Orozco-Terwengel et al., 2012], diffStat [Turner et al., 2011], association statistic [Turner and Miller, 2012] and the F_{ST} [Remolina et al., 2012]. We evaluated the performance of these statistics using truncating selection (as described above) where we retained 60% of the individuals with the highest phenotypic values. The R package ROCR was used to create the ROC curves [Sing et al., 2005].

Stabilizing selection and diminishing returns epistasis

We obtained 205 haplotypes from the DGRP lines (Freeze 2.0) and the recombination rate of *D. melanogaster* [Mackay et al., 2012, Comeron et al., 2012]. Beneficial alleles were introduced into four genes known to confer starvation resistance in Drosophila [gene (effect size): pnt (0.32), kekkon-1 (1.3), Tre1 (0.25), CG151 (0.78) [Harbison et al., 2004]]. The effect sizes was roughly proportional to the observed effect of the genes to starvation resistance [Harbison et al., 2004]. For both stabilzing selection and diminishing return epistasis the phenotypic value was mapped to fitness using a fitness function. For stabilizing selection we used a normal distribution with $\mu = -5$, $\sigma^2 = 2$, a minimum fitness of $f_{min} = 0.5$ and a maximum fitness of $f_{max} = 1.5$. For diminishing returns epistasis we used $f = f_{min} + (f_{max} - f_{min}) * (1 - \frac{1}{e^{\alpha(x+\beta)}})$, with $f_{min} = 0.5$, $f_{max} = 1.5$, $\alpha = 0.4$ and $\beta = 0.0$. We used a female to male ratio of 50:50 and a hemizygous X-chromosome. Simulations were performed for 40 generations and 10 replicates were used. Selected loci were identified with the cmh-test [Kofler et al., 2011] and Manhattan plots were generated with R .

Theoretical expectations used in validations

Genetic drift

To estimate the expected allele frequency after 50 generations of genetic drift in a population of size $N = 250$ we used a Markov chain model. We computed the transition probabilities among all allele frequencies using the binomial formula

[Gillespie, 2010], resulting in a 501×501 matrix. The expected allele frequency was computed by multiplying the starting frequency (50%) with the matrix 50 times.

Trajectory of selected locus

A trajectory of a selected locus with two alleles (A and a) can be estimated based on the fitness of each genotype [Gillespie, 2010]:

$$W_{AA} = 1 + s \tag{1}$$
$$W_{Aa} = 1 + hs \tag{2}$$
$$W_{aa} = 1 \tag{3}$$

where s is the selection coefficient, h is the heterozygous effect and W_{AA}, W_{Aa},W_{aa} the fitness of individuals with genotypes AA, Aa and aa respectively.

The allele frequency of a selected locus at time t can be estimated by:

$$p_t = \frac{p_{t-1}(p_{t-1}W_{AA} + q_{t-1}W_{Aa})}{\bar{w}}$$

where $t-1$ is the previous generation, p is the allele frequency of A, q is the allele frequency of a and $\bar{w}$ is the mean fitness of the population.

The mean fitness of the population can be estimated by:

$$\bar{w} = p^2 W_{AA} + 2pqW_{Aa} + q^2 W_{aa}$$

Response to selection

According to Falconer [1960] the expected response to selection on a quantitative trait can be estimated using the breeder's equation:

$$R = h^2 S$$

where h^2 is the heritability and S is the selection differential.

The selection differential can be estimated for truncating selection if the phenotypic values of the population follow a normal distribution.

$$S = i\sigma_P$$

where i is the selection intensity and σ_P is the phenotypic standard deviation of the population.

The selection intensity can be estimated by:

$$i = \frac{z}{p}$$

where z is the height of the ordinate at the point of truncation and p is the proportion of individuals surviving truncation selection.

Linkage disequilibrium decay

The correct implementation of recombination was validated by measuring the decay of LD over the course of 200 generations. The D value was used as an estimation of the LD between two loci. The expected D was calculated by:

$$D_t = D_0(1 - c)^t$$

where t is the generations, D_t, is the LD at generation t, D_0, is the initial LD and c is the recombination rate.

Benchmarking different tools using the DGRP data

MimicrEE2

Simulations with MimicrEE2 were performed with the DGRP haplotypes, the recombination rate of *D. melanogaster* and the following command:

```
java -jar mim2-v193.jar qt --haplotypes-g0
mimicree_format_dgrp_snps_X_chromosome
--recombination-rate comeron_sex_specific
--effect-size effect_size_1locus_X
--heritability 1 --snapshots 40
--replicate-runs 10
--output-sync starvation_resistance_sex_second.sync
--threads 8 --selection-regime ../selection_regime_08 --sex sex-info
```

The input files can be found here: https://sourceforge.net/projects/mimicree2/files/publication_files/

MimicrEE

MimicrEE1, does not support the simulation of quantitative traits and we thus performed neutral simulations. We used the DRGP haplotypes, the recombination rate of *D. melanogaster* and the following command:

```
java -jar MimicrEESummary.jar --haplotypes-g0
   mimicree_format_dgrp_snps_X_chromosome --recombination-rate
   dmel.rr.txt --output-mode 40 --replicate-runs 10 --output-format
   sync --output-file mimicree1_test --threads 8
```

The input files can be found here: https://sourceforge.net/projects/mimicree2/files/publication_files/

forqs

The DGRP haplotypes were converted into the ms format supported by forqs using a custom Python script. Recombination rate estimates were transformed into the HapMap format. When multiple replicates are simulated with forqs the input haplotypes must be repeated 10 times in the same haplotype file. This is a very time and memory consuming approach that largely affects the performance of forqs. We performed two simulations with forqs, in the first the output was allele frequencies and in the second the output was haplotypes.

We used the following forqs configuration file to estimate allele frequencies:

```
PopulationConfigGenerator_ConstantSize pcg
    chromosome_pair_count = 5
    population_size = 205
    population_count = 10
    generation_count = 40
    chromosome_lengths = 23011544 21146708 24543557 27905053 22422827
    fitness_function = fitness

LocusList qtl_loci_2L
        chromosome:position = 1 12822160

LocusList qtl_loci_3L
        chromosome:position = 3 16143897

LocusList qtl_loci_3R
        chromosome:position = 4 23345117

LocusList qtl_loci_X
        chromosome:position = 5 5679736

LocusList neutral_loci_2L
        chromosome:position = 1 4998
        chromosome:position = 1 5002
        chromosome:position = 1 5039
        chromosome:position = 1 5040
        chromosome:position = 1 5092
        chromosome:position = 1 5095
        chromosome:position = 1 5153
                   ...
                   ...
                   ...
        chromosome:position = 5 22422249
        chromosome:position = 5 22422279
        chromosome:position = 5 22422323
        chromosome:position = 5 22422335

VariantIndicator_File my_variant_indicator
        msfile = forqs_dgrp_haps_10pops
        loci=qtl_loci_2L qtl_loci_3L qtl_loci_3R qtl_loci_X
        neutral_loci_2L neutral_loci_2R neutral_loci_3L
        neutral_loci_3R neutral_loci_X

RecombinationPositionGenerator_RecombinationMap rpg
        filename=2L_rr_forqs
        filename=2R_rr_forqs
        filename=3L_rr_forqs
        filename=3R_rr_forqs
        filename=X_rr_forqs

Distribution_Constant positive_effect_2L
    value = 1.3

Distribution_Constant positive_effect_3L
    value = 0.78

Distribution_Constant positive_effect_3R
```

```
    value = 0.32

Distribution_Constant positive_effect_X
    value = 0.25

Distribution_Constant no_dominance
    value = 0

QTLEffectGenerator generator_positive_2L
    locus_list = qtl_loci_2L
    effect_size_distribution = positive_effect_2L
    dominance_distribution = no_dominance

QTLEffectGenerator generator_positive_3L
    locus_list = qtl_loci_3L
    effect_size_distribution = positive_effect_3L
    dominance_distribution = no_dominance

QTLEffectGenerator generator_positive_3R
    locus_list = qtl_loci_3R
    effect_size_distribution = positive_effect_3R
    dominance_distribution = no_dominance

QTLEffectGenerator generator_positive_X
    locus_list = qtl_loci_X
    effect_size_distribution = positive_effect_X
    dominance_distribution = no_dominance

QuantitativeTrait_IndependentLoci qt
    qtl_effect_generator =generator_positive_2L
    qtl_effect_generator =generator_positive_3L
    qtl_effect_generator =generator_positive_3R
    qtl_effect_generator =generator_positive_X
    environmental_variance = 0.0

FitnessFunction_TruncationSelection fitness
    quantitative_trait = qt
    proportion_selected = 0.8

Reporter_AlleleFrequencies reporter_allele_frequencies
    quantitative_trait = qt
    locus_list=neutral_loci_2L
    locus_list=neutral_loci_2R
    locus_list=neutral_loci_3L
    locus_list=neutral_loci_3R
    locus_list=neutral_loci_X

SimulatorConfig
    seed = 0
    output_directory = output_example_truncating
    population_config_generator = pcg
    variant_indicator = my_variant_indicator
    quantitative_trait = qt
    quantitative_trait = fitness
    reporter = reporter_allele_frequencies
    recombination_position_generator = rpg
```

We used the following forqs configuration file to track haplotype chunks:

```
PopulationConfigGenerator_ConstantSize pcg
    chromosome_pair_count = 5
    population_size = 205
    population_count = 10
    generation_count = 40
    chromosome_lengths = 23011544 21146708 24543557 27905053 22422827
    fitness_function = fitness


LocusList qtl_loci_2L
        chromosome:position = 1 12822160

LocusList qtl_loci_3L
        chromosome:position = 3 16143897

LocusList qtl_loci_3R
        chromosome:position = 4 23345117

LocusList qtl_loci_X
        chromosome:position = 5 5679736


LocusList neutral_loci_2L
        chromosome:position = 1 4998
        chromosome:position = 1 5002
        chromosome:position = 1 5039
                 ...
             ...
             ...
    chromosome:position = 5 22422249
        chromosome:position = 5 22422279
        chromosome:position = 5 22422323
        chromosome:position = 5 22422335


VariantIndicator_File my_variant_indicator
        msfile = forqs_dgrp_haps_10pops
        loci=qtl_loci_2L qtl_loci_3L qtl_loci_3R
        qtl_loci_X neutral_loci_2L neutral_loci_2R
        neutral_loci_3L neutral_loci_3R neutral_loci_X

RecombinationPositionGenerator_RecombinationMap rpg
        filename=2L_rr_forqs
        filename=2R_rr_forqs
        filename=3L_rr_forqs
        filename=3R_rr_forqs
        filename=X_rr_forqs




Distribution_Constant positive_effect_2L
    value = 1.3

Distribution_Constant positive_effect_3L
    value = 0.78
```

```
Distribution_Constant positive_effect_3R
    value = 0.32

Distribution_Constant positive_effect_X
    value = 0.25

Distribution_Constant no_dominance
    value = 0

QTLEffectGenerator generator_positive_2L
    locus_list = qtl_loci_2L
    effect_size_distribution = positive_effect_2L
    dominance_distribution = no_dominance

QTLEffectGenerator generator_positive_3L
    locus_list = qtl_loci_3L
    effect_size_distribution = positive_effect_3L
    dominance_distribution = no_dominance

QTLEffectGenerator generator_positive_3R
    locus_list = qtl_loci_3R
    effect_size_distribution = positive_effect_3R
    dominance_distribution = no_dominance

QTLEffectGenerator generator_positive_X
    locus_list = qtl_loci_X
    effect_size_distribution = positive_effect_X
    dominance_distribution = no_dominance

QuantitativeTrait_IndependentLoci qt
    qtl_effect_generator =generator_positive_2L
    qtl_effect_generator =generator_positive_3L
    qtl_effect_generator =generator_positive_3R
    qtl_effect_generator =generator_positive_X
    environmental_variance = 0.0



FitnessFunction_TruncationSelection fitness
    quantitative_trait = qt
    proportion_selected = 0.8

Reporter_Population haps_reporter

SimulatorConfig
    seed = 0
    output_directory = output_example_truncating
    population_config_generator = pcg
    variant_indicator = my_variant_indicator
    quantitative_trait = qt
    quantitative_trait = fitness
    reporter = haps_reporter
    recombination_position_generator = rpg
```

The haplotype identifiers at generation 40 were mapped onto the initial genomes to produce the new haplotypes using the following configuration file to perform simulations for one replicate:

```
# ms_map_config.txt
#
# sample mapping config file for forqs_map_ms
#


ms_filenames = forqs_dgrp_haps_10pops


chromosome = 1
position_begin = 0
position_end = 119029689


map_entry = 0 410 0 0
map_entry = 410 820 0 0
map_entry = 820 1230 0 0
map_entry = 1230 1640 0 0
map_entry = 1640 2050 0 0
map_entry = 2050 2460 0 0
map_entry = 2460 2870 0 0
map_entry = 2870 3280 0 0
map_entry = 3280 3690 0 0
map_entry = 3690 4100 0 0
```

quantiNemo

Since quantiNemo does not support simulating truncating selection for a quantitative trait we simulated random genetic drift instead. Genotypic data from the DGRP lines were converted to the FSTAT format using a custom Python script. We used a distance between consecutive loci of 0.1cM. Time estimates were obtained only for one run and then multiplied by the number of replicates (10). When simulations performed for ten replicates, memory usage was increasing to 300GB as quantiNemo stores the output of each generation in memory. The result was the interruption of the simulation process when the available memory was exhausted.

We used the following configuration file:

```
patch_number    1
patch_capacity  205
replicates      1
generations     40

ntrl_loci       3954651
ntrl_all        2
ntrl_ini_genotypes      qnhaps_dgrp_all

ntrl_loci_positions     $genetic_map_for_dgrp

stat    {n.adlt.nbAll}

stat_dir        stats
stat_save       0
```

```
stat_log_time   40
```

SliM2

The DGRP haplotypes were converted into the SliM2 format using a custom Python script. Initially we tried to convert an MS file containing the DGRP haplotypes into the SliM2 format. We terminated the process after 5 days of no result. SliM2 performs faster when the input file provided is in binary format. Simulations for multiple replicates are not possible with SliM2. Time estimates were obtained only for one run and then multiplied by the number of replicates (10). First we converted the input file into the binary format and and finally the following script was used for the simulations:

```
initialize() {
initializeSLiMOptions(mutationRuns=256);
initializeMutationRate(0.0);

initializeMutationType("m1", 0.5, "f", 0.0);
initializeMutationType("m2", 0.5, "f", 0.0);

initializeGenomicElementType("g1", m1, 1);
initializeGenomicElementType("g2", m1, 1);
initializeGenomicElementType("g3", m1, 1);
initializeGenomicElementType("g4", m1, 1);
initializeGenomicElementType("g5", m1, 1);

initializeGenomicElement(g1, 0, 23011543);
initializeGenomicElement(g2, 23011544, 44158251);
initializeGenomicElement(g3, 44158252, 68701808);
initializeGenomicElement(g4, 68701809, 96606861);
initializeGenomicElement(g5, 96606862, 119029689);

lines2L = readFile("/Users/vetgrid06/runslim/2L_rr_slim");
rates2L = NULL;
ends2L = NULL;
for (line2L in lines2L)
{
components2L = strsplit(line2L, "\t");
ends2L = c(ends2L, asInteger(components2L[0]));
rates2L = c(rates2L, asFloat(components2L[1]));
}
ends2L = c(ends2L[1:(size(ends2L)-1)] - 2, 23011543);
rates2L = rates2L * 1e-8;

lines2R = readFile("/Users/vetgrid06/runslim/2R_rr_slim");
rates2R = NULL;
ends2R = NULL;
for (line2R in lines2R)
{
components2R = strsplit(line2R, "\t");
ends2R = c(ends2R, asInteger(components2R[0])+23011543);

rates2R = c(rates2R, asFloat(components2R[1]));
}
ends2R = c(ends2R[1:(size(ends2R)-1)] - 2, 44158251);
```

```
rates2R = rates2R * 1e-8;

lines3L = readFile("/Users/vetgrid06/runslim/3L_rr_slim");
rates3L = NULL;
ends3L = NULL;
for (line3L in lines3L)
{
components3L = strsplit(line3L, "\t");
ends3L = c(ends3L, asInteger(components3L[0])+44158251);

rates3L = c(rates3L, asFloat(components3L[1]));
}

ends3L = c(ends3L[1:(size(ends3L)-1)] - 2, 68701808);
rates3L = rates3L * 1e-8;

lines3R = readFile("/Users/vetgrid06/runslim/3R_rr_slim");
rates3R = NULL;
ends3R = NULL;
for (line3R in lines3R)
{
components3R = strsplit(line3R, "\t");
ends3R = c(ends3R, asInteger(components3R[0])+68701808);

rates3R = c(rates3R, asFloat(components3R[1]));
}

ends3R = c(ends3R[1:(size(ends3R)-1)] - 2, 96606861);
rates3R = rates3R * 1e-8;

linesX = readFile("/Users/vetgrid06/runslim/X_rr_slim");
ratesX = NULL;
endsX = NULL;
for (lineX in linesX)
{
componentsX = strsplit(lineX, "\t");
endsX = c(endsX, asInteger(componentsX[0])+96606861);

ratesX = c(ratesX, asFloat(componentsX[1]));
}

endsX = c(endsX[1:(size(endsX)-1)] - 2, 119029689);
ratesX = ratesX * 1e-8;

initializeRecombinationRate(c(rates2L,0.5,rates2R,0.5,rates3L,0.5,rates3R
,0.5,ratesX), c(ends2L,23011544,ends2R,44158252,ends3L,68701809,
ends3R,96606862,endsX));
// Recombination map is finished

//return substitutions
m1.convertToSubstitution = F;
m2.convertToSubstitution = F;

//positions of non-neutral loci
defineConstant("Q2L", 12822159);
defineConstant("Q3L", 60302148);
defineConstant("Q3R", 92046925);
```

```
defineConstant("QX", 102286597);
}
//recombination map is finished

1 late() {
sim.readFromPopulationFile("/Users/vetgrid06/runslim/dgrp_slim_final_bin");
}
1 late(){
g=p1.genomes;
genome_vector1_pos=c(g[0:1], g[2:3],
g[4:5], g[6:7], g[8:9], g[10:11],
g[12:13], g[14:15], g[16:17], g[18:19],
g[20:21], g[24:25], g[30:31], g[32:33],
g[34:35], g[36:37], g[38:39], g[40:41],
g[42:43], g[44:45], g[46:47], g[50:51],
g[52:53], g[54:55], g[56:57], g[58:59],
g[60:61], g[62:63], g[64:65], g[68:69],
g[70:71], g[72:73], g[74:75], g[76:77],
g[78:79], g[80:81], g[82:83], g[84:85],
g[86:87], g[88:89], g[92:93], g[96:97],
g[98:99], g[100:101], g[102:103], g[104:105],
g[114:115], g[116:117], g[122:123],g[124:125],
g[126:127], g[128:129], g[132:133], g[134:135],
g[136:137], g[140:141], g[142:143], g[150:151],
g[152:153], g[154:155], g[156:157],g[158:159],
g[160:161], g[166:167], g[168:169], g[170:171],
g[172:173], g[174:175], g[176:177], g[180:181],
g[182:183], g[184:185], g[186:187], g[188:189],
g[190:191],g[192:193], g[194:195], g[196:197],
g[198:199], g[200:201], g[202:203], g[204:205],
g[208:209],g[210:211], g[212:213], g[214:215],
g[216:217],g[218:219], g[220:221], g[224:225],
g[226:227],g[228:229], g[230:231], g[232:233],
g[234:235], g[238:239], g[240:241], g[242:243],
g[244:245], g[246:247], g[248:249], g[250:251],
g[252:253], g[254:255], g[258:259], g[260:261],
g[262:263], g[266:267], g[268:269], g[270:271],
g[272:273], g[274:275], g[276:277], g[280:281],
g[282:283], g[284:285], g[286:287], g[292:293],
g[294:295], g[296:297], g[298:299], g[300:301],
g[302:303], g[304:305], g[306:307], g[308:309],
g[310:311], g[312:313], g[314:315], g[316:317],
g[318:319], g[320:321], g[322:323], g[324:325],
g[326:327], g[328:329], g[330:331], g[332:333],
g[334:335], g[336:337], g[338:339], g[340:341],
g[344:345], g[346:347], g[348:349], g[350:351],
g[352:353], g[354:355], g[356:357], g[358:359],
g[360:361], g[364:365], g[366:367], g[368:369],
g[372:373], g[374:375], g[376:377], g[378:379],
g[380:381], g[382:383], g[384:385], g[386:387],
g[388:389], g[390:391], g[392:393], g[394:395],
g[406:407], g[408:409]);

genome_vector1_neg=c(g[22:23], g[26:27], g[28:29],
g[48:49], g[66:67], g[90:91], g[94:95],
g[106:107], g[108:109], g[110:111],
g[112:113], g[118:119], g[120:121],
```

```
g[130:131], g[138:139], g[144:145],
g[146:147], g[148:149], g[162:163],
g[164:165], g[178:179], g[206:207],
g[222:223], g[236:237], g[256:257],
g[264:265], g[278:279], g[288:289],
g[290:291], g[342:343], g[362:363],
g[370:371], g[396:397], g[398:399],
g[400:401], g[402:403], g[404:405]);


genome_vector2_pos=c(g[0:1], g[2:3], g[4:5], g[6:7],
g[8:9], g[12:13], g[14:15], g[16:17], g[18:19],
g[20:21], g[22:23], g[24:25], g[26:27],
g[28:29], g[30:31], g[32:33], g[34:35],
g[36:37], g[40:41], g[44:45], g[46:47],
g[48:49], g[50:51], g[52:53], g[54:55],
g[56:57], g[58:59], g[60:61], g[62:63],
g[64:65], g[66:67], g[68:69], g[70:71],
g[72:73], g[76:77], g[78:79], g[80:81],
g[82:83], g[84:85], g[86:87], g[88:89],
g[90:91], g[92:93], g[94:95], g[96:97],
g[98:99], g[100:101], g[102:103], g[104:105],
g[106:107], g[110:111], g[112:113], g[114:115],
g[116:117], g[118:119], g[120:121], g[122:123],
g[124:125], g[128:129], g[130:131], g[134:135],
g[136:137], g[138:139], g[140:141], g[142:143],
g[144:145], g[146:147], g[148:149], g[152:153],
g[154:155], g[158:159], g[160:161], g[164:165],
g[166:167], g[170:171], g[172:173], g[174:175],
g[178:179], g[180:181], g[182:183], g[184:185],
g[186:187], g[188:189], g[190:191], g[192:193],
g[194:195], g[196:197], g[198:199], g[200:201],
g[202:203], g[204:205], g[206:207], g[210:211],
g[214:215], g[216:217], g[218:219], g[220:221],
g[222:223], g[226:227], g[228:229], g[230:231],
g[232:233], g[234:235], g[236:237], g[238:239],
g[240:241], g[242:243], g[244:245], g[248:249],
g[250:251], g[254:255], g[256:257], g[260:261],
g[262:263], g[264:265], g[266:267], g[268:269],
g[270:271], g[272:273], g[274:275], g[276:277],
g[278:279], g[280:281], g[282:283], g[284:285],
g[286:287], g[288:289], g[290:291], g[292:293],
g[294:295], g[298:299], g[300:301], g[302:303],
g[304:305], g[306:307], g[310:311], g[312:313],
g[314:315], g[316:317], g[318:319], g[320:321],
g[322:323], g[324:325], g[326:327], g[328:329],
g[334:335], g[336:337], g[338:339], g[340:341],
g[342:343], g[346:347], g[352:353], g[354:355],
g[358:359], g[360:361], g[362:363], g[366:367],
g[368:369], g[370:371], g[372:373], g[374:375],
g[378:379], g[380:381], g[382:383], g[386:387],
g[388:389], g[390:391], g[392:393], g[394:395],
g[396:397], g[398:399], g[400:401], g[402:403],
g[406:407], g[408:409]);

genome_vector2_neg=c(g[10:11], g[38:39], g[42:43],
g[74:75], g[108:109], g[126:127], g[132:133],
```

```
g[150:151], g[156:157], g[162:163], g[168:169],
g[176:177], g[208:209], g[212:213], g[224:225],
g[246:247], g[252:253], g[258:259], g[296:297],
g[308:309], g[330:331], g[332:333], g[344:345],
g[348:349], g[350:351], g[356:357], g[364:365],
g[376:377], g[384:385], g[404:405]);

genome_vector3_pos=c(g[0:1], g[2:3], g[4:5], g[6:7],
g[8:9], g[12:13], g[14:15], g[16:17], g[18:19],
g[20:21], g[22:23], g[24:25], g[26:27], g[30:31],
g[32:33], g[34:35], g[36:37], g[42:43], g[44:45],
g[46:47], g[50:51], g[54:55], g[56:57], g[58:59],
g[62:63], g[64:65], g[70:71], g[74:75], g[78:79],
g[80:81], g[82:83], g[84:85], g[86:87], g[88:89],
g[92:93], g[94:95], g[96:97], g[98:99], g[100:101],
g[102:103], g[106:107], g[112:113], g[114:115],
g[120:121], g[122:123], g[124:125], g[130:131],
g[132:133], g[136:137], g[138:139], g[140:141],
g[142:143], g[144:145], g[146:147], g[148:149],
[150:151], g[158:159], g[160:161], g[162:163],
g[164:165], g[166:167], g[168:169], g[170:171],
g[174:175], g[176:177], g[178:179], g[180:181],
g[182:183], g[188:189], g[190:191], g[194:195],
g[196:197], g[198:199], g[200:201], g[204:205],
g[206:207], g[208:209], g[210:211], g[214:215],
g[216:217], g[222:223], g[226:227], g[232:233],
g[234:235], g[236:237], g[238:239], g[242:243],
g[252:253], g[258:259], g[260:261], g[262:263],
g[264:265], g[268:269], g[270:271], g[272:273],
g[274:275], g[276:277], g[278:279], g[280:281],
g[282:283], g[284:285], g[290:291], g[292:293],
g[294:295], g[296:297], g[298:299], g[300:301],
g[302:303], g[304:305], g[308:309], g[310:311],
g[312:313], g[318:319], g[320:321], g[326:327],
g[328:329], g[330:331], g[332:333], g[334:335],
g[336:337], g[338:339], g[340:341], g[342:343],
g[346:347], g[348:349], g[352:353], g[356:357],
g[360:361], g[362:363], g[364:365], g[366:367],
g[370:371], g[372:373], g[374:375], g[376:377],
g[382:383], g[384:385], g[386:387], g[388:389],
g[390:391], g[392:393], g[394:395], g[396:397],
g[398:399], g[400:401], g[404:405], g[408:409]);

genome_vector3_neg=c(g[10:11], g[28:29], g[38:39],
g[40:41], g[48:49], g[52:53], g[60:61], g[66:67],
g[68:69], g[72:73], g[76:77], g[90:91], g[104:105],
g[108:109], g[110:111], g[116:117], g[118:119],
g[126:127], g[128:129], g[134:135], g[152:153],
g[154:155], g[156:157], g[172:173], g[184:185],
g[186:187], g[192:193], g[202:203], g[212:213],
g[218:219], g[220:221], g[224:225], g[228:229],
g[230:231], g[240:241], g[244:245], g[246:247],
g[248:249], g[250:251], g[254:255], g[256:257],
g[266:267], g[286:287], g[288:289], g[306:307],
g[314:315], g[316:317], g[322:323], g[324:325],
g[344:345], g[350:351], g[354:355], g[358:359],
g[368:369], g[378:379], g[380:381], g[402:403], g[406:407]);
```

```
genome_vector4_pos=c(g[0:1], g[2:3], g[4:5], g[6:7],
g[12:13], g[24:25], g[28:29], g[32:33], g[36:37],
g[38:39], g[44:45], g[46:47], g[48:49], g[52:53],
g[54:55], g[58:59], g[60:61], g[62:63], g[64:65],
g[66:67], g[68:69], g[70:71], g[72:73], g[76:77],
g[78:79], g[82:83], g[84:85], g[86:87], g[88:89],
g[90:91], g[92:93], g[96:97], g[100:101], g[106:107],
g[108:109], g[110:111], g[112:113], g[114:115],
g[118:119], g[122:123], g[124:125], g[128:129],
g[130:131], g[134:135], g[136:137], g[140:141],
g[144:145], g[146:147], g[148:149], g[150:151],
g[160:161], g[162:163], g[166:167], g[174:175],
g[176:177], g[180:181], g[188:189], g[190:191],
g[192:193], g[194:195], g[198:199], g[204:205],
g[206:207], g[208:209], g[210:211], g[212:213],
g[214:215], g[216:217], g[218:219], g[220:221],
g[222:223], g[228:229], g[230:231], g[232:233],
g[234:235], g[236:237], g[240:241], g[244:245],
g[246:247], g[250:251], g[252:253], g[258:259],
g[262:263], g[264:265], g[268:269], g[270:271],
g[272:273], g[278:279], g[280:281], g[282:283],
g[286:287], g[288:289], g[292:293], g[296:297],
g[298:299], g[300:301], g[302:303], g[304:305],
g[306:307], g[308:309], g[310:311], g[312:313],
g[320:321], g[328:329], g[332:333], g[336:337],
g[338:339], g[340:341], g[344:345], g[354:355],
g[364:365], g[368:369], g[372:373], g[386:387],
g[388:389], g[390:391], g[392:393], g[398:399],
g[400:401], g[402:403]);

genome_vector4_neg=c(g[8:9], g[10:11], g[14:15], g[16:17],
g[18:19], g[20:21], g[22:23], g[26:27], g[30:31],
g[34:35], g[40:41], g[42:43], g[50:51], g[56:57],
g[74:75], g[80:81], g[94:95], g[98:99], g[102:103],
g[104:105], g[116:117], g[120:121], g[126:127],
g[132:133], g[138:139], g[142:143], g[152:153],
g[154:155], g[156:157], g[158:159], g[164:165],
g[168:169], g[170:171], g[172:173], g[178:179],
g[182:183], g[184:185], g[186:187], g[196:197],
g[200:201], g[202:203], g[224:225], g[226:227],
g[238:239], g[242:243], g[248:249], g[254:255],
g[256:257], g[260:261], g[266:267], g[274:275],
g[276:277], g[284:285], g[290:291], g[294:295],
g[314:315], g[316:317], g[318:319], g[322:323],
g[324:325], g[326:327], g[330:331], g[334:335],
g[342:343], g[346:347], g[348:349], g[350:351],
g[352:353], g[356:357], g[358:359], g[360:361],
g[362:363], g[366:367], g[370:371], g[374:375],
g[376:377], g[378:379], g[380:381], g[382:383],
g[384:385], g[394:395], g[396:397], g[404:405],
g[406:407], g[408:409]);

//estimate phenotypic values of individuals
genome_vector1_pos.addNewMutation(m2, 1.3, Q2L);
genome_vector1_neg.addNewMutation(m2, -1.3, Q2L);
```

```
genome_vector2_pos.addNewMutation(m2, 0.78, Q3L);
genome_vector2_neg.addNewMutation(m2, -0.78, Q3L);

genome_vector3_pos.addNewMutation(m2, 0.32, Q3R);
genome_vector3_neg.addNewMutation(m2, -0.32, Q3R);

genome_vector4_pos.addNewMutation(m2, 0.25, QX);
genome_vector4_neg.addNewMutation(m2, -0.25, QX);

}

fitness(m2){
return 1.0;
}

late(){
inds = sim.subpopulations.individuals;

phenotypes=inds.sumOfMutationsOfType(m2);

inds.tag = 1;

x=order(phenotypes);

tokill=x[0:40];

inds[tokill].tag = 0;
}

//perform truncation selection

fitness(NULL) {
// individuals tagged for death die here
if (individual.tag == 1)
return 1.0;
else
return 0.0;
}


//return output after 40 generations
1 late() {

cat("--------------\n");

cat("Generation 1, Frequencies and positions of mutations of type m1:\n\n");

cat(sim.mutationFrequencies(p1,sim.mutationsOfType(m1))+
"\t"+sim.mutationsOfType(m1).position+"\n");
print('\n');
cat("Generation 1, Frequencies and positions of mutations of type m2:\n\n");
cat(sim.mutationFrequencies(p1,sim.mutationsOfType(m2))+
"\t"+sim.mutationsOfType(m2).position+"\n");

print('\n');
cat("Generation 1 is finished");
print('\n');
```

```
print('\n');

}

40 late(){
cat("Generation 40, Frequencies of mutations of type m1:\n\n");
cat(sim.mutationFrequencies(p1,sim.mutationsOfType(m1))+
"\t"+sim.mutationsOfType(m1).position+"\n");

print('\n');
cat("Generation 40, Frequencies of mutations of type m2:\n\n");


cat(sim.mutationFrequencies(p1,sim.mutationsOfType(m2))+
"\t"+sim.mutationsOfType(m2).position+"\n");
print('\n');
cat("Generation 40 is finished");
print('\n');


}
```

FFPopSim

A Python script was used to generate 410 haploid individuals and 3954651 SNPs which then used as input to perform truncation selection simulations with FFPopSim. Time estimates were obtained only for one run and then multiplied by the number of replicates (10). In the final time estimation we did not take into account the time required to generate the new haplotypes but rather only the actual running time of the simulator. Notice that after running the script the number of generations returned from the simulator will be 121. Actually the real number is 40, as the number of generations increases every time we update the genotypes of the population. Here we simulate truncation selection, and we update the population genotypes 3 times per generation (120/3=40).

```
import numpy as np
from matplotlib import pyplot as plt
import FFPopSim as h

from optparse import OptionParser, OptionGroup
import random
import timeit
from itertools import chain



start=timeit.default_timer()




parser = OptionParser()
parser.add_option("--haps",dest="haps",help="Number of random haps")
parser.add_option("--snps",dest="snps",help="Number of random snps")
```

```
(options, args) = parser.parse_args()


haps=[]
total_haps=[]
counts=[]
truncated_genotypes=[]
for i in range(0,int(options.haps)):

    for j in range(0, int(options.snps)):

        number=random.random()
        if number<=0.50:
            haps.append(0)
        else:
            haps.append(1)

    total_haps.append(haps)
    haps=[]
    counts.append(1)

stop = timeit.default_timer()
print "Elapsed time (sec):", (stop - start)


#print total_haps
#print counts


L=int(options.snps)
N=int(options.haps)
pop=h.haploid_highd(L)
pop.carrying_capacity = int(options.haps)
pop.crossover_rate=  0.05
pop.outcrossing_rate = 0


print pop.generation

pop.set_genotypes(total_haps,counts)

print  "Generation:", pop.generation

maxgen=121
print pop.status()
print "\n"






print "BASE POPULATION"
selection_coefficients = 0.0*np.ones(pop.L)
sc=[1000000,400000,1900000,3000000]
tp = [pop.generation]
```

```
for i in sc:
        selection_coefficients[i] = 0.1

pop.set_trait_additive(selection_coefficients,0)

print pop.get_genotypes()
print pop.get_fitnesses()

print "\n"




print "TRUNCATED BASE POPULATION"
#####base population######
sorted_fitness=sorted((round(num,4),ind) for ind,num in enumerate(pop.get_fitnesses()))
truncated_fitness=sorted_fitness[82:len(sorted_fitness)]


for i,n in enumerate(truncated_fitness):
        index_to_keep=n[1]
        truncated_genotypes.append(pop.get_genotype(index_to_keep)*1)

pop.set_genotypes(truncated_genotypes,[1]*len(truncated_fitness))

print pop.get_genotypes()
print pop.get_fitnesses()
#####base population######

#print pop.generation





while pop.generation<maxgen:
        for i in sc:
                selection_coefficients[i] = 0.0

        pop.set_trait_additive(selection_coefficients,0)



        print "##########"
        ind_fit=[]
        genos=[]
        ind_genotype=[]
        truncated_genotypes=[]


        print pop.get_genotypes()
        print pop.get_fitnesses()
        print pop.get_clone_sizes()

        print "##########"

        pop.evolve(1)
```

```
print "Evolution function is called here"

for i in sc:
        selection_coefficients[i] = 0.1

pop.set_trait_additive(selection_coefficients ,0)

print "\n"

print pop.get_genotypes()
print pop.get_fitnesses()
print pop.get_clone_sizes()


for i in range(0, len(pop.get_fitnesses())):
        fitness= pop.get_fitness(i)
        ind_fit.append([fitness]*pop.get_clone_size(i))
        genotypes=[pop.get_genotype(i)]*pop.get_clone_size(i)
        ind_genotype.append(genotypes)

ind_fit= list(chain.from_iterable(ind_fit))

for i in range(0, len(ind_genotype)):
        for j in ind_genotype[i]:
                genos.append(j*1)


pop.set_genotypes(genos ,[1]*pop.population_size)


print pop.get_genotypes()
print pop.get_fitnesses()
print pop.get_clone_sizes()

sorted_fitness=sorted((round(num ,4),ind) for ind,num in enumerate(pop.get_fitnesses())
truncated_fitness=sorted_fitness[82:len(sorted_fitness)]


for i,n in enumerate(truncated_fitness):
        index_to_keep=n[1]
        truncated_genotypes.append(pop.get_genotype(index_to_keep)*1)


pop.set_genotypes(truncated_genotypes ,[1]*len(truncated_fitness))

print sorted_fitness
print pop.get_genotypes()
print pop.get_fitnesses()
print pop.get_clone_sizes()


tp.append(pop.generation)

print "\n"
print "\n"
print pop.generation
```

```
print "\n"

pop.status()


stop2 = timeit.default_timer()

print "Elapsed time (sec):", (stop2 - stop)
```

References

J.M. Comeron, R. Ratnappan, and S. Bailin. The Many Landscapes of Recombination in *Drosophila melanogaster*. *PLoS Genetics*, 8(10):e1002905, 2012.

Douglas Scott Falconer. *Introduction to quantitative genetics.* Oliver And Boyd; Edinburgh; London, 1960.

John H Gillespie. *Population genetics: a concise guide.* JHU Press, 2010.

Susan T. Harbison, Akihiko H. Yamamoto, Juan J. Fanara, Koenraad K. Norga, and Trudy F.C. Mackay. Quantitative Trait Loci Affecting Starvation Resistance in *Drosophila melanogaster*. *Genetics*, 166(4):1807–1823, 2004.

Robert Kofler, Ram Vinay Pandey, and Christian Schlötterer. PoPoolation2: identifying differentiation between populations using sequencing of pooled DNA samples (Pool-Seq). *Bioinformatics (Oxford, England)*, 27(24):3435–6, 2011.

TF Mackay, S Richards, EA Stone, A Barbadilla, JF Ayroles, D Zhu, S Casillas, Y Han, MM Magwire, JM Cridland, MF Richardson, RR Anholt, M Barrón, C Bess, KP Blankenburg, MA Carbone, D Castellano, L Chaboub, L Duncan, Z Harris, M Javaid, JC Jayaseelan, SN Jhangiani, KW Jordan, F Lara, F Lawrence, SL Lee, P Librado, RS Linheiro, AJ Lyman, RF nad Mackey, M Munidasa, DM Muzny, L Nazareth, I Newsham, L Perales, LL Pu, C Qu, M Ràmia, JG Reid, SM Rollmann, J Rozas, N Saada, L Turlapati, KC Worley, YQ Wu, A Yamamoto, Y Zhu, CM Bergman, KR Thornton, D Mittelman, and Gibbs RA. The Drosophila Melanogaster genetic reference panel. *Nature*, 482(7384):173–178, 2012.

Pablo Orozco-Terwengel, Martin Kapun, Viola Nolte, Robert Kofler, Thomas Flatt, and Christian Schlötterer. Adaptation of Drosophila to a novel laboratory environment reveals temporally heterogeneous trajectories of selected alleles. *Molecular Ecology*, 21(20):4931–4941, 2012.

Silvia C Remolina, Peter L Chang, Jeff Leips, Sergey V Nuzhdin, and Kimberly A Hughes. Genomic basis of aging and life-history evolution in Drosophila melanogaster. *Evolution; international journal of organic evolution*, 66(11):3390–403, 2012.

Tobias Sing, Oliver Sander, Niko Beerenwinkel, and Thomas Lengauer. ROCR: visualizing classifier performance in R. *Bioinformatics (Oxford, England)*, 21 (20):3940–1, 2005.

Thomas L Turner and Paige M Miller. Investigating natural variation in Drosophila courtship song by the evolve and resequence approach. *Genetics*, 191(2):633–42, 2012.

T.L. Turner, Andrew D., Stewart Andrew, T. Fields, William R. Rice, and Aaron M. Tarone. Population-Based Resequencing of Experimentally Evolved Populations Reveals the Genetic Basis of Body Size Variation in Drosophila melanogaster. *PLoS Genetics*, 7(3):e1001336, 2011.
